# Smoking prevalence among pregnant women from 2007 to 2012 at a tertiary-care hospital

**DOI:** 10.1007/s00431-016-2710-1

**Published:** 2016-03-18

**Authors:** Angelika Schultze, Herbert Kurz, Ingrid Stümpflen, Erich Hafner

**Affiliations:** Medical University of Vienna, Spitalgasse 23, 1090 Wien, Austria; Departement of Pediatrics, SMZ Ost Hospital, Langobardenstraße 122, 1220 Wien, Austria; Department of Obstetrics and Gynecology at SMZ Ost Hospital, Langobardenstraße 122, 1220 Wien, Austria

**Keywords:** Smoking prevalence, Pregnancy, Small for gestational age, Maternal characteristics, Vienna

## Abstract

The harmful effects of smoking during pregnancy are well known, but we lack prevalence data concerning this subject in Austria. The aim ofz the present study was to determine the prevalence and any changes in the prevalence of smoking during pregnancy in the last few years. The investigation was conducted at a perinatal center in Vienna, Austria. Further aims of the study were to evaluate maternal characteristics associated with smoking and demonstrate the harmful effects of smoking on neonatal outcome in this population. Once inquired, self-reported smoking during pregnancy, maternal age, and neonatal data from 2007 to 2012 were evaluated retrospectively. Of birth records, 11,142 were analyzed. From 2007 to 2012, the prevalence of smoking declined significantly from 19.1 to 15.6 %. The overall prevalence was 18.1 % and was highest (43.7 %) among young women (<20 years). The risk of small for gestational age (SGA) was significantly higher among newborns of smoking mothers.

*Conclusion*: The prevalence of smoking among pregnant women has declined in Austria in the last few years but is still quite high. Prevention programs should focus on young women, who are at highest risk in this regard.

**Table:** 

**What is Known:** • *Smoking during pregnancy is known to exert harmful effects*
**What is New:** • *Paucity of epidemiological data regarding this subject in Austria* • *Significant decline of self-reported smoking during pregnancy from 2007 to 2012 in Vienna*

## Introduction

Although the harmful effects of smoking during pregnancy are well known and have been investigated for more than 50 years, a significant number of women continue to smoke during pregnancy. Thus, smoking still is a public health concern. In addition to causing the fetal tobacco syndrome [10], it has been held responsible for the stagnation of infant mortality in the last decade in Austria, along with alcohol consumption and obesity in pregnant women [26]. Secondary complications due to maternal smoking lead to higher hospital admission rates of infants from the postnatal phase to the age of 5 years, which signifies higher health-care-related costs [2, 16].

## Smoking in the general population in Austria

With a smoking prevalence of 34 % in 2009, Austria ranks among the top six countries in the European Union [23], assumes the fourth highest rank in daily smoking (26 % of adult women smoked on a daily basis in 2008) [17], and is among the countries with the highest prevalence of teenage smoking. The overall prevalence of teenage smoking is 25.4 %; among girls, it ranges from 21.1 to 30.4 % depending on the age group [9].

## Smoking during pregnancy in Europe and Austria

A study comprising data from six European countries (Belgium, Bulgaria, Germany, Greece, Ireland, and Portugal) showed that the prevalence of smoking at the time of delivery varied between 7 % (Bulgaria) and 52.5 % (Ireland), with a mean smoking prevalence of 19.6 % [8]. Twenty-one percent of pregnant women smoked in Germany [18], while 20–30 % of pregnant women smoked in Austria in 2010 [10]. A study conducted in Spain in 2013, addressing the effects of smoking on fetal biometry, comprised 2478 women and revealed an overall smoking prevalence of 32 % [4]. These data are consistent with similar studies in Europe.

## Harmful effects of smoking on the fetus

Smoking exerts harmful effects in every stage of pregnancy. It is the most important preventable risk factor for an adverse pregnancy outcome [1, 11]. Horak et al. [10] summarized the known effects as the fetal tobacco syndrome. Cigarette smoke contains toxins in quantities that affect placental and fetal cell growth, proliferation, and differentiation and exert harmful effects on the development of organ systems including the cardiovascular, respiratory, and nervous system. Smoking reduces blood flow to the placenta and accumulates carboxyhemoglobin in the fetus, both of which cause chronic hypoxic stress for the fetus [1]. Placental metabolism is altered in smoking women, and their placenta also revealed structural differences as well as oxidative damage of the placental tissue [21]. These effects might explain the higher risk of miscarriage, fetal growth restriction, stillbirth, preterm birth, and placenta praevia [11]. Fetal growth restriction results in small for gestational age neonates. These neonates are burdened with both short- and long-term health effects, including higher risk for hyperglycemia during the neonatal period, failing to thrive during childhood, and obesity, early-onset type 2 diabetes, and arterial hypertension during adulthood [19].

## Aims of the study

The aims of the study were the following:

1. To determine a statistically significant change, if any, in the prevalence of smoking (yes/no) among pregnant women from 2007 to 2012.
2. To elucidate risk factors for maternal smoking, specifically whether maternal age and parity influenced its prevalence. Besides, neonatal outcome data were analyzed to confirm the harmful effects of maternal smoking on newborns and determine whether changes in prevalence would be reflected in neonatal parameters.

## Patients and Methods

The data for the study were collected at the obstetric department of Sozialmedizinisches Zentrum (SMZ) Ost, a tertiary-care medical center providing a wide spectrum of mother/child health care. Data were collected from 2007 to 2012. Every woman with a singleton pregnancy, who had delivered her child after the 22nd week of gestation, was included in this study. The women’s general and pregnancy-related medical history was registered when they booked their delivery at the hospital. Their current consumption of tobacco (yes/no; if yes number of cigarettes/day) was registered in a personal conversation by a midwife or a doctor and entered in an electronic data sheet. Women were classified into (0) non-smokers or (1) smokers; and the numbers of cigarettes per day were documented. Smoking behavior was registered once during pregnancy. Maternal age was divided into the following categories: (0) younger than 20 years, (1) 20–25 years, (2) 26–30 years, (3) 31–36 years, and (4) over 36 years. Neonatal characteristics included birthweight and weight percentile. The duration of pregnancy in weeks and days was also registered. The children were classified as preterm births (37 + 0 weeks or below) or small for gestational age (below the 10th percentile). Women who gave birth more than once in the study period were treated as separate individuals in the respective study year. Data obtained from a survey conducted in 2002, comprising 576 women in the identical hospital setting, were used to compare the prevalence of smoking. All data were pseudonymized.

## Statistical analysis

Basic patient and neonatal data were collected at the initial exploratory data analysis. Multiple logistic regressions were used (a) to explore the association between the year of documentation and the occurrence of smoking, (b) to assess upward and downward trends in the association between smoking during pregnancy and maternal age, (c) to test for the occurrence of *small for gestational age* children in connection with maternal smoking, and (d) to test for the age distribution throughout the study years. Linear regression was used to assess the association between maternal age and the number of cigarettes smoked per day. The *χ*^2^ test was used to test for significance as to whether parity is associated with smoking during pregnancy. The test was also used to determine the significance of relative risk and the number of cigarettes causing preterm births and *small for gestational age* newborns.

Kendall’s correlation coefficient was used to determine whether smoking behavior changed between pregnancies. The analysis was performed with IBM SPSS Statistics 21. The relative risk and the number of cigarettes needed to cause harm were calculated by using a modified Microsoft Excel sheet by Dr. Georg Heinze available at http://www.meduniwien.ac.at/user/georg.heinze/mb2. The level of significance was set to *p* < 0.05.

## Results

Of 12,302 women who underwent their first antenatal investigation at SMZ Ost Hospital, 11,142 were included in the study. Of the records, 1160 were excluded because of twin/triplet pregnancies (*n* = 823 single records), missing information in the charts, not completing the 22nd week of gestation, or being lost to follow-up (*n* = 337).

## Characteristics of women and newborns

In the final dataset, 2022 (18.1 %) women had smoked during the study years. The mean age of non-smokers was 30.33 years (SD = 5.574) and that of smokers was 27.56 years (SD = 5.983). A total of 394 women were below 20 years of age, of whom 222 (1.9 %) were non-smokers and 172 (1.5 %) smokers. The largest non-smoking group was 31–36 years old (34.5 %) and the largest smoking group 20–25 years old (32.1 %). The mean number of cigarettes smoked per day was 8.84 (SD = 5.268), which corresponded to the category of 5–10 cigarettes per day (43.9 %). Of the smokers, 36.2 % smoked 1–5 cigarettes per day, and 19.9 % smoked more than 10 cigarettes per day. Parity was rather evenly distributed among both groups, with slightly more primiparous women in both groups. The mean duration of pregnancy in weeks was slightly higher in the non-smoking group (38.85 % [SD = 2.384] vs. 38.69 % [SD = 2.47]; Table 1).

**Table 1:** Maternal characteristics

Characteristics of women	Non-smokers	Smokers
*n* (%)	9120 (81.9 %)	2022 (18.1 %)
Year of delivery		
2007	1441 (80.9 %)	341 (19.1 %)
2008	1503 (80.7 %)	360 (19.3 %)
2009	1490 (80.7 %)	357 (19.3 %)
2010	1479 (81.5 %)	336 (18.5 %)
2011	1593 (82.8 %)	330 (17.2 %)
2012	1614 (84.4 %)	298 (15.6 %)
Age (mean, standard deviation)	30.33 ± 5.574	27.56 ± 5.983
Age categories	Non-smokers	Smokers
Younger than 20 years	222 (2.4 %^a^, 1.9 %^c^)	172 (8.5 %^b^, 1.5 %^c^)
20–25 years	1606 (17.6 %^a^, 14.4 %^c^)	649 (32.1 %^b^, 5.8 %^c^)
26–30 years	2837 (31.1 %^a^, 25.5 %^c^)	581 (28.7 %^b^, 5.2 %^c^)
31–36 years	3149 (34.5 %^a^, 28.5 %^c^)	448 (22.2 %^b^, 4.2 %^c^)
Over 36 years	1306 (14.3 %^a^, 11.7 %^c^)	172 (8.5 %^b^, 1.5 %^c^)
Number of cigarettes smoked per day (mean, standard deviation)	0	8.84 ± 5.268
Number of cigarettes smoked per day	Non-smokers	Smokers
0 cigarette/day	9120	0 (81.9 %)
1–5 cigarettes/day	0	732 (36.2 %^b^, 6.6 %^c^)
5–10 cigarettes/day	0	887 (43.9 %^b^, 7.9 %^c^)
More than 10 cigarettes/day	0	403 (19.9 %^b^, 3.6 %^c^)
Parity	Non-smokers	Smokers
	4329 (47.5 %^a^, 38.9 %^c^)	915 (45.3 %^b^, 8.2 %^c^)
	4791 (52.5 %^a^, 42.9 %^c^)	1107 (54.7 %^b^, 9.9 %^c^)
Primiparous		
Multiparous		
Duration of pregnancy in weeks	38.85 ± 2.384	38.69 ± 2.47

^a^Within smokers

^b^Within non-smokers

^c^Within study population

To determine changes in smoking behavior between pregnancies, women who had delivered more than one baby at the hospital during the study period were viewed separately for the purpose of the investigation. Of 1255 women who had two babies at the study hospital, 212 smoked during one or both pregnancies. Seventy-six (35.8 %) women increased their cigarette consumption, 43 (20.3 %) smoked the same number of cigarettes, and the remaining women (*n* = 93; 43.9 %) reduced or quit smoking. Of 45 women who had three babies at the hospital, 15 had smoked during one or more pregnancies.

Data concerning the newborns (Table 2) were divided according to maternal smoking behavior. The infants of non-smoking women had a higher mean birthweight in grams (3352.7 [SD = 615.57] vs. 3158 [SD = 604.36]). The mean weight percentile of infants born to smokers was 34.57 (SD = 25.49) and that of non-smokers was 43.94 (SD = 26.91). Preterm birth rates were 8.3 % among non-smoking women and 9.5 % among smoking women. The incidence of *small for gestational age* children was slightly higher among smokers than among non-smokers,

**Table 2:** Characteristics of newborns

Neonatal outcome	Non-smokers	Smokers
Weight in grams (mean, standard deviation)	3352.7 ± 615.57	3158.39 ± 604.36
Weight percentile (mean, standard deviation)	43.94 ± 26.91	34.57 ± 25.49
	Non-smokers	Smokers
Preterm births (*n*, %)	758 (8.3 %^a^, 6.8 %^c^)	194 (9.5 %^b^, 1.7 %^c^)
	Non-smokers	Smokers
Small for gestational age (*n*, %)	1037 (11.4 %^a^, 9.3 %^c^)	347 (17.2 %^b^, 3.1 %^c^)

^a^Within non-smokers

^b^Within smokers

^c^Within study population

## Risk factors for maternal smoking

**Fig. 1:**
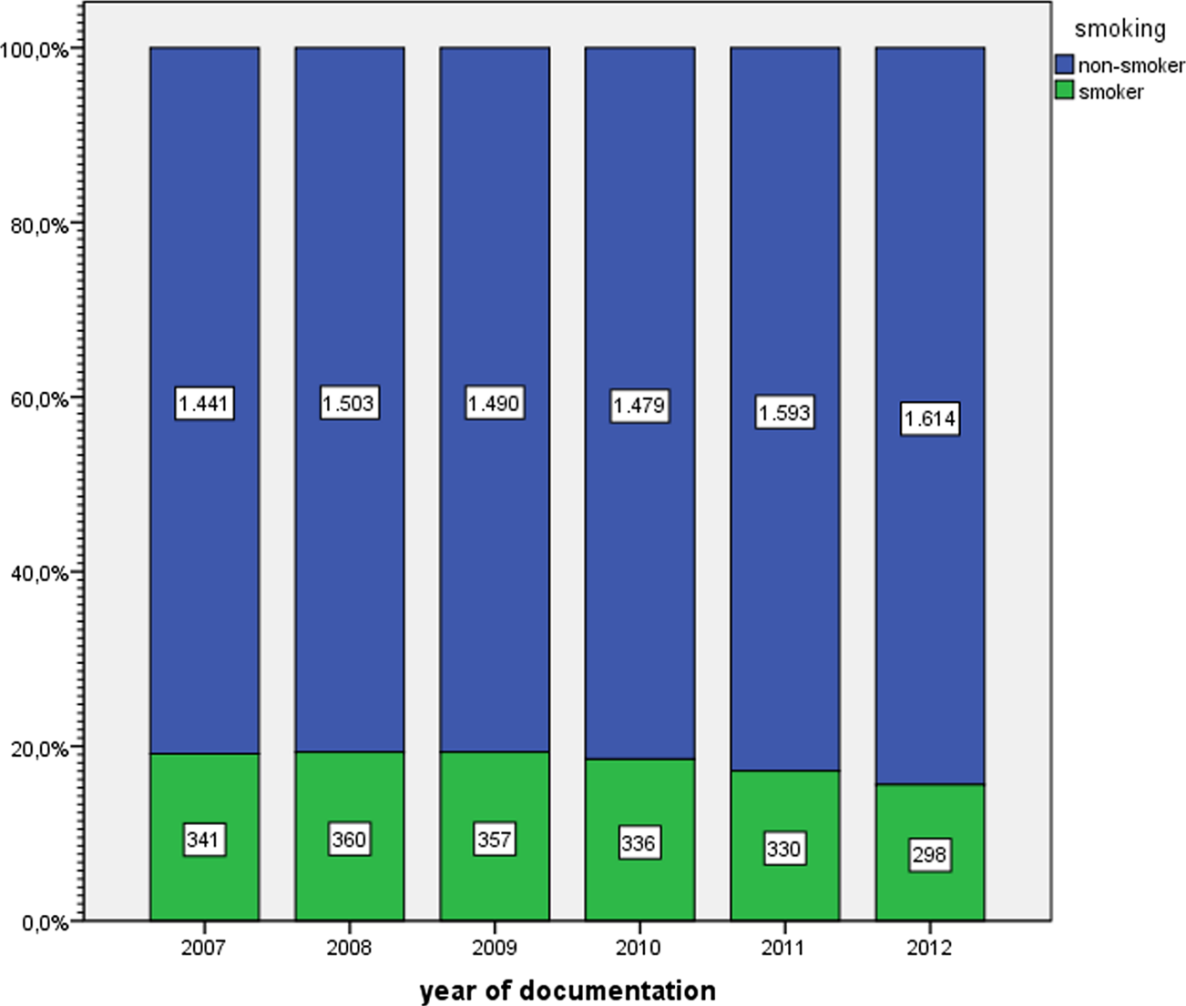
Change of maternal smoking in the study years in percent and absolute numbers

The overall percentage of smokers during the study years was 18.1 %, increased from 19.1 % in 2007 to 19.3 % in 2008, then remained constant at 19.3 % for 1 year and fell steadily thereafter, reaching the lowest figure of 15.6 % in 2012. The decline was significant in the logistic regression analysis with an odds ratio (OR) of 0.952 (95 % confidence interval (CI) 0.926–0.980, *p* = 0.00; Fig. 1).

The age distribution of pregnant women did not change significantly throughout the study years (−0.22, 95 % CI −0.46–1.52, *p* = 0.42).

**Fig. 2:**
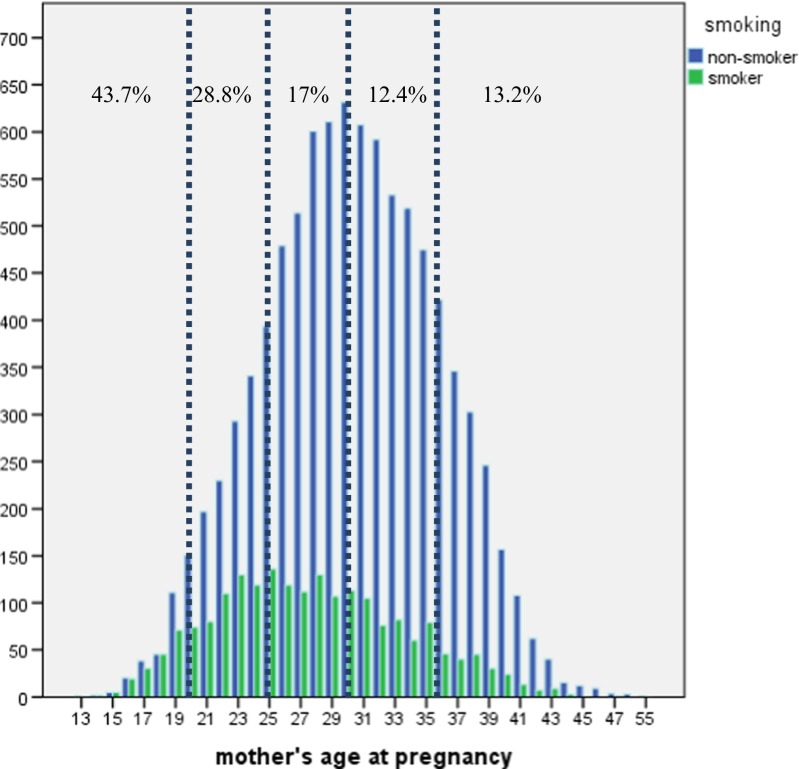
Maternal age and smoking

With regard to the age distribution, smokers accounted for 43.7 % of women younger than 20 years and 28.8 % of those aged 20–25 years. The percentage of maternal smoking decreased with age; the lowest percentage of 13.2 % was observed in women older than 36 years of age (Fig. 2). The overall logistic regression function showed that the prevalence of smoking fell steadily with increasing maternal age; the OR was 0.917 (95 % CI 0.908–0.925, *p* = 0.00).

Linear regression, which only took smokers (*n* = 2022) into account, revealed no linear relationship between maternal age and cigarette consumption. The model showed a *R*^2^ and adjusted *R*^2^ of 0.01, with a standard error of estimate of 5.266 (*p* = 0.00). The weak *R*^2^ clearly indicates the absence of a linear relationship between the two variables.

With regard to the number of births and smoking during pregnancy, the *χ*^2^ test revealed a significant decline in smoking with increasing numbers of births (31.359 [critical value 18.307], *df* = 10, *p* = 0.001).

Kendall’s correlation coefficient *τb* was used to assess changes in smoking behavior between pregnancies. With a correlation coefficient *τb* of 0.365 (*p* = 0.00), no strong correlation was registered between smoking during the first and the second pregnancies. The analyses of three consecutive pregnancies revealed a moderately positive correlation (*τb* = 0.611, *p* = 0.00) between the first and the second pregnancies but an insignificant weak positive correlation between the second and the third pregnancies (*τb* 0.394, *p* = 0.106).

## Neonatal outcome

Of 952 preterm infants, 194 (20.4 %) were born to smoking mothers. In the risk analysis, the risk of bearing preterm infants was just slightly higher among smokers than among non-smoking women (9.6 vs. 8.3 %). The relative risk was 1.15 (95 % CI 0.99–1.34), and the number of cigarettes needed to cause harm was 12.9 (95 % CI −832.4–37.2). The *χ*^2^ test for 1 *df* was 3.49 (*p* = 0.0619).

Of 1424 small for gestational age (SGA) children, 27.2 % (*n* = 387) were born to smoking mothers. In the risk analysis, the risk of bearing an SGA infant was 19.21 % (95 % CI 17.51–21) for smokers in contrast to 11.42 % for non-smokers (95 % CI 10.78–12.10). Thus, the relative risk of a smoker having a low-birthweight child increased by 1.68 (95 % CI 1.51–1.87), and the number of cigarettes needed to cause harm was 12.9 (95 % CI 10.4–16.38). This becomes significant in the *χ*^2^ test, with a value of 89.23 for 1 *df* (*p* < 0.0001).

The number of SGA children did not change statistically in the study period. The odds ratio for the decline was 1.033 (95 % CI 0.966–1.041, *p* = 0.887) among smokers and 1.033 (95 % CI 0.968–1.104, *p* = 0.328) among non-smokers.

## Discussion

This study, conducted at a tertiary perinatal center in Vienna, revealed that 18.1 % of pregnant women smoked during pregnancy; 19.1 % smoked in 2007, the highest prevalence of 19.3 % was registered in 2008/2009, and a steady decline was observed thereafter, culminating in 15.6 % at the end of the study period in 2012. A survey conducted in 2002, comprising 576 pregnant women at the same hospital, had shown that 21.5 % smoked during pregnancy (unpublished data). Thus, the prevalence of smoking a few years before the commencement of the present investigation appears to have been even higher.

The most recent data in Austria—derived from 2010—show that 20–30 % of pregnant women smoked [10]. However, the rates registered in the present study were consistently lower than 20 %. Although maternal smoking has been decreasing in other countries over the last few years [5, 25] and a steady decline was also noted in the present investigation, smoking rates remain high among young mothers below 20 years of age (43.7 %) and those aged 20–25 years (28.8 %). In our population, smoking rates among women older than 36 years were below the overall prevalence.

The high prevalence of smoking among very young pregnant women has been attributed to the high-risk behavior and lack of responsibility at this age, which might have led to pregnancy in the first place [7, 12]. This may well be true in view of the fact that the smoking prevalence among pregnant women below the age of 20 years, registered in the present study, was approximately 20 % higher than that in the general population.

The present study revealed no association between age and the number of cigarettes smoked per day. The authors of previous studies mention that heavy smokers were largely older mothers because of their prolonged exposure to tobacco before pregnancy and therefore greater addiction to nicotine [9, 15]; both of these studies comprised much larger sample sizes.

We registered a reduction in smoking rates during pregnancy with the number of births. A positive correlation was noted between smoking during the first and second pregnancies but not between the second and third pregnancies. This might indicate rising awareness of the harmful effects of smoking on the part of the 15 women who experienced three pregnancies during the study period. However, it may also have been due to underreporting, secondary to social stigma experienced in previous pregnancies.

Although the present study did indicate a higher risk of preterm births in conjunction with smoking, the increased risk was not statistically significant. This may have been due to the sample size; the majority of studies addressing preterm births and maternal smoking comprised much larger sample sizes [3, 6, 13].

SGA is another birth outcome related to maternal smoking during pregnancy [24]. The present study clearly showed a higher risk of SGA among smoking mothers; the risk was 7.8 % higher and the number of cigarettes needed to cause harm (in respect of giving birth to an SGA child) was 12.9.

Although maternal smoking clearly declined in the study period (19.1 to 15.6 %), the decline in SGA children was not statistically significant. This may have been due to the transfer of high-risk pregnancies to the perinatal center and their impact on birth outcome statistics. Another explanation could be that smoking, in fact, did not decrease and the lower figures indirectly reflected underreporting in the last few years. As there have been no intensive campaigns denouncing smoking in Austria and smoking is commonly regarded as a habit rather than a health-damaging phenomenon, the underreporting thesis seems unlikely. Currently, we have no data concerning changes in underreporting rates.

The strength of the present study is its large sample size; 11,142 complete maternal records comprising self-reported smoking behavior, maternal characteristics, and neonatal outcome were studied. The most relevant limitation is that the information on smoking status is based on self-reporting alone. This may have led to underreporting because of the stigma associated with smoking, especially during pregnancy. Furthermore, underreporting may have been common in women who had experienced previous pregnancies with unfavorable outcomes and had been told that smoking cessation may prevent problems in the forthcoming pregnancy. Although a number of authors registered rather high discrepancies in self-reported smoking status and cotinine measurement [22], many of them agree that self-reporting is a reliable means of determining smoking status during pregnancy [14]. In a Spanish study [4], the authors observed accurate self-reporting (3.9 % misreporting) and a very similar overall prevalence of smoking as in the present study (18.5 %).

Another limitation of the present study is that no socioeconomic data were collected. However, given the fact that the entire investigation was performed in a single region and the same hospital setting, socioeconomic factors would probably have not changed considerably during the study years. In contrast to many countries, the absence of social security or access to prenatal and perinatal care is no hindrance in Austria; 98 % of the population have excellent social security coverage [20]. The high density of hospitals providing prenatal and perinatal care and the “mother-and-child card” (Mutter-Kind-Pass) is an established social security measure. This mother-and-child card includes free but mandatory examinations during pregnancy and until the child’s second birthday. The first mandatory examination must be completed before the 16th gestational week. This checkup is taken quite seriously because of its ensuing health benefits and child support payment.

A further limitation of the study is that the women’s smoking status was only documented once during pregnancy. For various reasons—such as the woman’s condition being graded as a high-risk pregnancy some time later during her pregnancy—this documentation was not performed at the same time point in all women. The time of documentation varied from the first trimester to the late third trimester. However, most of the information was obtained from early pregnancy. Data concerning the cessation of smoking were not registered. A meta-analysis by the CDC showed high variability in cessation rates [25]. The present study did not address smoking cessation, although cessation rates would probably have not altered the results substantially.

## Conclusion

Once inquired, self-reported smoking during pregnancy declined from 19.1 to 15.6 % in the study years. Urine cotinine was not measured. Although the prevalence of smoking among young women is still alarmingly high, the largest number of smoking women is in the 22–31-year age category. Hence, both age groups should be the focus of attention of anti-smoking campaigns targeted at pregnant women. Primary educational programs should focus on the youth in order to prevent addiction at an early age. Further educational programs, including the use of social media and online courses, should be implemented.

## Abbreviations

CI: Confidence interval
HBSC: Health behavior in school-aged children
NICU: Neonatal Intensive Care Unit
OB/Gyn: Obstetrics and gynecology
OR: Odds ratio
SD: Standard deviation
SGA: Small for gestational age
SMZ: Sozialmedizinisches Zentrum
WHO: World Health Organization

## References

[1] Abbott LC, Winzer-Serhan UH (2012). Smoking during pregnancy: lessons learned from epidemiological studies and experimental studies using animal models. Crit Rev Toxicol.

[2] Adams EK, Miller VP, Ernst C, Nishimura BK, Melvin C, Merritt R (2002). Neonatal health care costs related to smoking during pregnancy. Health Econ.

[3] Aliyu MH, Lynch O, Saidu R, Alio AP, Marty PJ, Salihu HM (2010). Intrauterine exposure to tobacco and risk of medically indicated and spontaneous preterm birth. Am J Perinatol.

[4] Aurrekoetxea JJ, Murcia M, Rebagliato M, López MJ, Castilla AM, Santa-Marina L, et al (2013) Determinants of self-reported smoking and misclassification during pregnancy, and analysis of optimal cut-off points for urinary cotinine: a cross-sectional study. BMJ Open. 3(1) 10.1136/bmjopen-2012-002034 PMC3563144 23355667

[5] Bergmann R, Bergmann K, Schumann S, Richter R, Dudenhausen J (2008). Rauchen in der Schwangerschaft: Verbreitung, Trend, Risikofaktoren. Z Für Geburtshilfe Neonatol.

[6] Burguet A, Kaminski M, Abraham‐Lerat L, Schaal J-P, Cambonie G, Fresson J (2004). The complex relationship between smoking in pregnancy and very preterm delivery. BJOG Int J Obstet Gynaecol.

[7] Egebjerg Jensen K, Jensen A, Nøhr B, Krüger KS (2008). Do pregnant women still smoke? A study of smoking patterns among 261,029 primiparous women in Denmark 1997–2005. Acta Obstet Gynecol Scand.

[8] Giersiepen K, Janssen B, Tsoneva-Pentcheva L. Euro-scip-III-survey: an international comparison of smoking prevalence in pregnant women based on a pooled analysis of data collected in six European countries [Internet]. [cited 2013 Jul 21]. Available from: http://www.womenofthenorthwest.net/newsletters/Bips%20Pregnancy%20and%20Smoking%20Research%20Report.pdf

[9] HBCS Factsheet Nr. 04/2012 [Internet]. Available from: http://bmg.gv.at/home/Schwerpunkte/Praevention/Schulgesundheit/WHO_Studie_Health_Behaviour_in_School_aged_Children_

[10] Horak F, Fazekas T, Zacharasiewicz A, Eber E, Kiss H, Lichtenschopf A (2012). The fetal tobacco syndrome—a statement of the Austrian Societies for General- and Family Medicine (ÖGAM), Gynecology and Obstetrics (ÖGGG), Hygiene, Microbiology and Preventive Medicine (ÖGHMP), Pediatrics and Adolescence Medicine (ÖGKJ) as well as Pneumology (ÖGP). Wien Klin Wochenschr.

[11] Jauniaux E, Burton GJ (2007). Morphological and biological effects of maternal exposure to tobacco smoke on the feto-placental unit. Early Hum Dev.

[12] Kramer MS, Séguin L, Lydon J, Goulet L (2000). Socio‐economic disparities in pregnancy outcome: why do the poor fare so poorly?. Paediatr Perinat Epidemiol.

[13] Kyrklund-Blomberg NB, Granath F, Cnattingius S (2005). Maternal smoking and causes of very preterm birth. Acta Obstet Gynecol Scand.

[14] Kvalvik LG, Nilsen RM, Skjærven R, Vollset SE, Midttun Ø, Ueland PM (2012). Self-reported smoking status and plasma cotinine concentrations among pregnant women in the Norwegian mother and child cohort study. Pediatr Res.

[15] Mohsin M, Bauman AE (2005). Socio-demographic factors associated with smoking and smoking cessation among 426,344 pregnant women in New South Wales, Australia. BMC Public Health.

[16] Petrou S, Hockley C, Mehta Z, Goldacre M (2005). The association between smoking during pregnancy and hospital inpatient costs in childhood. Soc Sci Med.

[17] Rauchverhalten in Österreich [Internet]. Available from: http://www.api.or.at/akis/download/factsheetrauchverhalten.pdf

[18] Röske K, Lingnau M-L, Hannöver W, Haas J-P, Thyrian JR, Fusch C (2008). Prevalence of smoking in women before and during pregnancy: population-based data. Dtsch Med Wochenschr.

[19] Ross MG, Beall MH (2008). [Adult sequelae of intrauterine growth restriction] semin. Perinatology.

[20] Statistische Daten aus der Sozialverscherung-Monatsbericht August [Internet]. [cited 2013 Sep 23]. Available from: http://www.sozialversicherung.at/portal27/portal/esvportal/channel_content/cmsWindow?action=2&p_menuid=3738&p_tabid=2

[21] Sbrana E, Suter MA, Abramovici AR, Hawkins HK, Moss JE, Patterson L (2011). Maternal tobacco use is associated with increased markers of oxidative stress in the placenta. Am J Obstet Gynecol.

[22] Shipton D, Tappin DM, Vadiveloo T, Crossley JA, Aitken DA, Chalmers J (2009). Reliability of self reported smoking status by pregnant women for estimating smoking prevalence: a retrospective, cross sectional study. BMJ.

[23] Special eurobarometer 332, tobacco [Internet]. Available from: http://ec.europa.eu/health/tobacco/docs/ebs332_en.pdf

[24] Suzuki K, Sato M, Zheng W, Shinohara R, Yokomichi H, Yamagata Z (2014). Effect of maternal smoking cessation before and during early pregnancy on fetal and childhood growth. J Epidemiol Jpn Epidemiol Assoc.

[25] Tong VT, Dietz PM, Morrow B, D’Angelo DV, Farr SL, Rockhill KM, et al (2013) Trends in smoking before, during, and after pregnancy—pregnancy risk assessment monitoring system, United States, 40 sites, 2000–2010. Morb Mortal Wkly Rep Surveill Summ Wash DC 2002. Nov 8;62(6):1–19 24196750

[26] Waldhör T, Vutuc C, Haidinger G, Mittlböck M, Kirchner L, Wald M (2005). Trends in infant mortality in Austria between 1984 and 2002. Wien Klin Wochenschr.

